# Genome-Wide Epigenetic and Transcriptomic Characterization of Human-Induced Pluripotent Stem Cell–Derived Intestinal Epithelial Organoids

**DOI:** 10.1016/j.jcmgh.2018.10.008

**Published:** 2018-10-23

**Authors:** Judith Kraiczy, Alexander D.B. Ross, Jessica L. Forbester, Gordon Dougan, Ludovic Vallier, Matthias Zilbauer

**Affiliations:** 1 Department of Pediatrics, University of Cambridge, Cambridge, United Kingdom; 2 Wellcome Trust–MRC Cambridge Stem Cell Institute, Anne McLaren Laboratory and Department of Surgery, University of Cambridge, United Kingdom; 3 Wellcome Trust Sanger Institute, Wellcome Trust Genome Campus, Hinxton, Cambridge, United Kingdom; 4 Institute of Infection and Immunity, School of Medicine, Cardiff University, Heath Park, Cardiff, United Kingdom; 5 Department of Medicine, University of Cambridge, Cambridge, United Kingdom; 6 Department of Pediatric Gastroenterology, Hepatology and Nutrition, Addenbrooke’s Hospital, Cambridge, United Kingdom; 7 Wellcome Trust-Medical Research Council Stem Cell Institute, University of Cambridge, United Kingdom

**Keywords:** iPSC, induced pluripotent stem cell, iPSCo, induced pluripotent stem cell organoid, IEC, intestinal epithelial cell, SC, sigmoid colon, SCo, sigmoid colon organoid

Human induced pluripotent stem cells (iPSC) have been used to generate intestinal organoids that mimic key intestinal properties without the requirement for invasive procedures to obtain human tissues. The main protocols that have been described result in gut organoids that contain both intestinal epithelium as well as mesenchymal cells.^1^, ^2^ We have previously reported on human iPSC-derived intestinal organoids (iPSCo) that can be propagated in long-term culture that contain solely epithelial cells.^3^, ^4^, ^5^, ^6^, ^7^ A pure epithelial model offers unique opportunities to study epithelial cell intrinsic and cell-type–specific mechanisms. Among these cellular processes are epigenetic mechanisms such as DNA methylation, which acts as a key regulator of intestinal epithelial development and regional identity.^1^, ^7^ The purpose of this study was to characterize iPSCo by comparing these cultures with primary purified intestinal epithelial cells (IECs).

**Figure 1:**
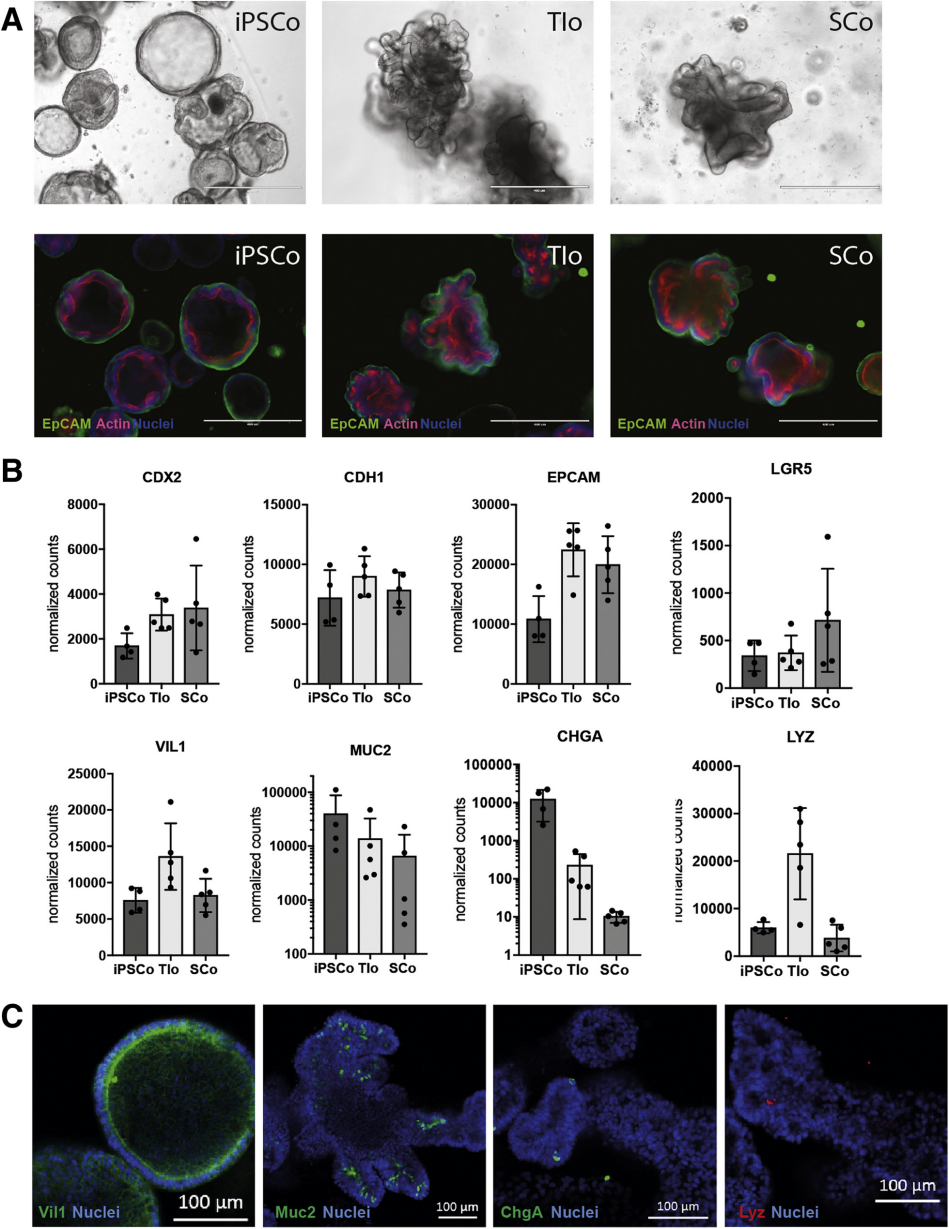
**Characterization of iPSC-derived intestinal epithelial organoids.** (*A*) Intestinal epithelial organoids derived from PSCs (iPSCo), terminal ileum organoids (TIo), or SC organoids (SCo) in microscopic brightfield view (*upper panel*), or immunofluorescent staining for epithelial cell adhesion molecule (epithelial cell adhesion molecule [EpCAM], green), actin (red), and nuclei (blue). *Scale bars*: 400 μm. (*B*) Expression of IEC-type marker as normalized counts from RNA sequencing data of the respective organoids. *Bar* shows means ± SD, n = 4–5 per group. (*C*) Immunofluorescent staining for enterocyte marker villin (VIL1, green), goblet cell marker mucin 2 (MUC2, green), enteroendocrine marker chromogranin A (CHGA, green), Paneth cell marker lysozyme (LYZ, red), and nuclear counterstain (blue), *Scale bars*: 100 um.

Intestinal epithelial organoid cultures were derived from at least 3 different lines of iPSCs according to the described protocol^3^, ^4^, ^5^ (see also Supplementary Figure 1), and showed a morphology comparable with mucosal biopsy-derived intestinal epithelial organoids. This was highlighted by the presence of epithelial cell adhesion molecules and epithelial polarization (Figure 1*A*). Furthermore, iPSCos were found to express several epithelial cell markers including E-cadherin (*CDH1*) and the intestinal stem cell marker *LGR5* (Figure 1*B*). Immunofluorescent staining showed ubiquitous positivity for the enterocyte marker villin, with specific subsets of cells expressing mucin 2, chromogranin A, and lysozyme, markers associated with the epithelial cell subsets Goblet, enteroendocrine, and Paneth cells, respectively (Figure 1*B* and *C*).

To further evaluate regional identity and the degree of developmental maturation of iPSCos, we performed DNA methylation and transcriptomic profiling using Illumina (Illumina, Cambridge, UK) Infinium bead arrays and RNA sequencing, respectively. We compared these genome-wide profiles with data sets we previously derived from purified IECs from mature terminal ileum and sigmoid colon (SC), as well as human fetal proximal gut and fetal distal gut (Supplementary Tables 1 and 2).^6^

**Figure 2:**
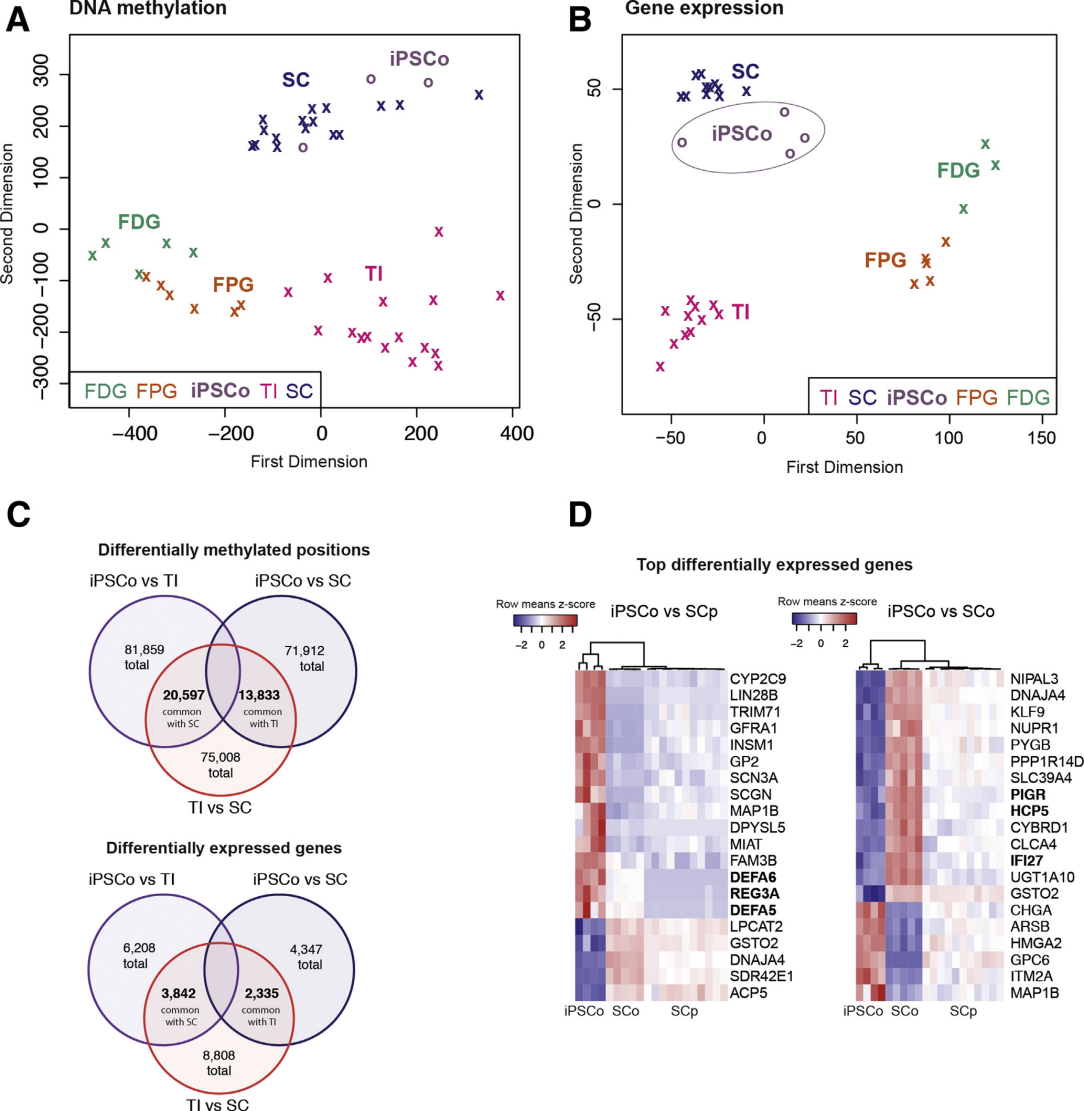
**Genome-wide profiling of human iPSCo and purified IECs.** (*A*) Multidimensional scaling plot showing sample similarity of genome-wide DNA methylation measured by an Illumina 450K array of iPSCo, in the context of IECs derived from terminal ileum (TI), SC, fetal proximal gut (FGP), and fetal distal gut (FDG).^1^ Each symbol represents a sample, x = IEC, o = organoid. (*B*) MDS plot of RNA-sequencing profiles showing the same sample groups as in panel *A*. Sample distance in the plot is based on regularized-logarithmic transformed read counts of all expressed genes. (*C*) Venn diagram indicating the number of differentially methylated CpG positions (*top*) or differentially expressed genes (*bottom*) (adjusted *P* < .01) between the different groups. (*D*) Heatmap of expression values (regularized-logarithmic normalized counts) of the top 20 most significantly differentially expressed genes comparing iPSCo and SC purified epithelium (SCp) (*left*), and iPSCo and SCo (*right*) for all 3 sample groups. The scale indicates the row mean Z-score (means, 0). The dendrogram above the heatmap clusters samples based on the expression similarity of those genes.

Relative sample similarity based on multidimensional scaling indicated that iPSCos clustered more closely to mature colonic epithelium, both on an epigenetic (ie, DNA methylation) and transcriptomic level (Figure 2*A* and *B*). This was confirmed further by performing unsupervised, hierarchical clustering (Supplementary Figure 2), as well as differential DNA methylation and gene expression analyses. The latter showed that iPSCos had a greater number of significantly differentially methylated positions (adjusted *P* < .01) and differentially expressed genes (adjusted *P* < .01) in common with the SC than with the terminal ileum (Figure 2*C*, Supplementary Table 3).

Given the overall similarity with pediatric sigmoid colon epithelium, we examined this relationship in more detail using mucosa-derived SC organoids (SCos) as an additional reference (Supplementary Figure 3*A* and *B*). We focused on the most significant differentially expressed genes (adjusted *P* < .01) between iPSCo and either purified IECs (SC purified epithelium) or organoids (SCo), respectively. Interestingly, some key small intestinal markers for Paneth cells (eg, α-defensins *DEFA5* and *DEFA6*) were abundant in iPSCo, while several genes involved in innate defense (eg, *IFI27*, *HCP5*, *PIGR*) showed markedly lower expression levels (Figure 2*D*). This indicated that iPSCos may not reach the full level of differentiation into small or large intestinal epithelium as found in vivo. Thus, iPSCo models may require further optimization of the differentiation protocol to permit further maturation and regionalization. Studying the signaling pathways that could drive this maturation could offer unique insight into the processes involved in intestinal development.

Together, our findings indicate that iPSC-derived human intestinal epithelial cell organoids more closely resemble mature colonic epithelium, which is in keeping with reports from other groups using alternative protocols to achieve distalization and maturation.^8^, ^9^, ^10^ However, using a genome-wide approach to validate the iPSCo model also uncovered distinct differences and incomplete regionalization of iPSC-derived organoids compared with human primary cells. These observations provide an ideal starting point to further investigate the factors required to model complete epithelial development in vitro.
