# Supplementary material for: Genome-Wide Epigenetic and Transcriptomic Characterization of Human-Induced Pluripotent Stem Cell–Derived Intestinal Epithelial Organoids
Source: Cell Mol Gastroenterol Hepatol. 2018 Oct 23;7(2):285–8. doi: 10.1016/j.jcmgh.2018.10.008 (PMC6354438; doi:10.1016/j.jcmgh.2018.10.008)
Supplement: Supplementary Material [file mmc1.pdf]

Supplementary Information

Methods

Human intestinal samples

All human tissue samples collected for this study were obtained following ethical approval and informed consent (REC-12/EE/0482, REC-17/EE/0265 and REC-96/085). Specifically, mucosal biopsies were collected from children between 5-15 years of age undergoing routine diagnostic endoscopy at Cambridge University Hospitals NHS Foundation Trust (Cambridge, UK). Only patients without underlying gastrointestinal pathology were included in the study. Fetal gut samples were obtained from elective terminations at 8-12-week gestation. Fetal gut was divided into the proximal (small intestine) and distal (large intestine) sections at the ileo-caecal junction.

Organoid culture

Intestinal epithelial organoids (IEO) were generated from mucosal biopsies using a protocol described previously [1], based on the original description of the approach [2].

Human induced pluripotent stem cells (hiPSC) were differentiated into IEO using a protocol described in Forbester et al [3–5]. The healthy control hiPSC lines Yemz1, Lise1 and Kolf2 were acquired through the Human Induced Pluripotent Stem Cells Initiative Consortium (HipSci www.hipsci.org). Two further hiPSC lines (13B and CA1ATD) were provided by LV[6].

Intestinal epithelial cells were purified from pediatric mucosal biopsies and fetal gut samples using enzymatic digestion and an autoMACS cell separation system (Miltenyi Biotec, Bergisch Gladbach, Germany) positively selecting for CD326 (i.e. EpCAM)-positive cells as described previously [7,8].

Immunofluorescent staining

Organoids in Matrigel (Corning, Corning, NY, USA) were fixed with 4% formaldehyde (Sigma Aldrich, St. Louis, MO, USA), washed with PBS and blocked for 3 hours in 10% goat serum (New England Biolabs, Hitchin, UK) +1% Bovine Serum Albumin +0.05% Tween-20 (both Sigma Aldrich). All antibodies were purchased from Abcam (Cambridge, UK). Antibodies against human EpCAM, Villin1, Chromogranin A, Mucin 2 and Lysozyme were applied after blocking

overnight at 4°C. After washing, secondary antibodies were used overnight at 4°C with added nuclear dye (DAPI, Sigma-Aldrich) or Phalloidin-Rhodamine (Biotium, Fremont, CA, USA). Brightfield and fluorescent images were obtained using an EVOS FL system (Life Technologies, Carlsbad, CA, USA) as well as a Zeiss LSM700 confocal microscope (Zeiss, Oberkochen, Germany).

DNA and RNA extraction, reverse transcription and real-time PCR

DNA and RNA were extracted with AllPrep Mini kit (Qiagen, Hilden, Germany) according to manufacturer's instructions. RNA was reverse transcribed using QuantiTect reverse transcription kit (Qiagen). Primer sequences are available upon request.

DNA was quantified using a QuBit dsDNA broad range kit (Thermo Fisher, Waltham, MA) and bisulfite-converted (Zymo Research, Irvine, CA) according to manufacturer's instructions.

Arrays and sequencing

DNA methylation was quantified using Illumina Infinium HumanMethylation450 BeadChip (Illumina, San Diego, CA) at the Genomics core at University College London (UCL) according to standard protocols, as described previously [1].

mRNA libraries were prepared following the Illumina TruSeq protocol (Illumina, Cambridge, UK) and sequenced on the Illumina HiSeq2500 platform at the Wellcome Trust Sanger Institute, as described previously [1].

Bioinformatic analyses

DNA methylation analysis

Processing including filtering (detection $P < 1 \times e^{-5}$), functional normalization, exclusion of X and Y chromosomes and probes co-locating with SNPS was performed using the Bioconductor package *minfi* [9]. DNA Methylation values were called using *minfi* [9]. DNA methylation levels can be quantified in Beta values (ranging from 0= Unmethylated to 1= Fully Methylated). M-values represent log-transformed Beta-values. M-values were used for analysis due to better statistical performance [10]. Package *Sva* [11] was applied for batch correction using the "ComBat" function. Multidimensional scaling (MDS) plots display relative sample similarity based on Euclidian distance on a 2-dimensional scale. The distance calculation included all CpG positions measured. MDS plots were based on M-values and drawn with *minfi* [9]. Differential

methylation analysis was performed on M-values using *limma* [12] with an adjusted *P <0.01.* Hierarchical clustering of samples (Supplementary Figure 2A) were based on M-values using Euclidian distance and agglomeration method "ward.D2".

RNA sequencing analysis

Processing of sequencing data included removal of low quality reads using *fastq_illumina_filter*, adapter trimming using *cutadapt* [13]. Filtered reads were mapped to GRCh37 with *tophat2* [14]*, bowtie* [15] and *samtools* [16]. Raw read alignments were counted using *htseq-count* [17]. Batch correction was performed based on expression of housekeeping genes using *RUVseq* [18]*.* Batch-corrected raw read counts were passed on to the *DESeq2* package [19]. Multidimensional scaling plots were based on normalized (i.e. regularized logarithm *(rlog*) – transformed counts obtained from *DESeq2* [19]. MDS-plots included all detected transcripts for sample distance calculation. Differential gene expression analysis was performed using *DESeq2* [19] and a cut-off of adjusted *P <0.01.*

Normalized counts (Figure 1B) were obtained from *DEseq2* using the "plotCounts" function and exported to GraphPad Prism 7 for graphical representation. Heatmaps were created on *rlog*-transformed counts, clustering samples by columns, using *heatmap3* [20]*.* Hierarchical clustering (Supplementary Figure 2B) was based on *rlog*-counts using Euclidian distance and agglomeration method "ward.D2".

Statistical analysis of overlapping positions (differentially expressed genes and differentially methylated positions) was performed in R using the representation factor and associated p-value as in Kim et al [21]

Data access

Profiling data of iPSC-derived organoids is available in ArrayExpress under accession E-MTAB-7289 (DNA methylation) and E-MTAB-7306 (RNA-sequencing). Previously published data is accessible for DNA methylation data under ArrayExpress accession E-MTAB-4957; for RNA sequencing under ArrayExpress accession E-MTAB-5015 or in the European Nucleotide Archive (ENA) Study PRJEB15114 [1].

All authors had access to all data and have reviewed and approved the final manuscript.

References

1. Kraiczy J, Nayak KM, Howell KJ, et al. DNA methylation defines regional identity of human intestinal epithelial organoids and undergoes dynamic changes during development. *Gut*. 2017;In press.:gutjnl-2017-314817. doi:10.1136/gutjnl-2017-314817.
2. Sato T, Stange DE, Ferrante M, et al. Long-term expansion of epithelial organoids from human colon, adenoma, adenocarcinoma, and Barrett's epithelium. *Gastroenterology*. 2011;141(5):1762-1772. doi:10.1053/j.gastro.2011.07.050.
3. Forbester JL, Hannan N, Vallier L, Dougan G. Derivation of Intestinal Organoids from Human Induced Pluripotent Stem Cells for Use as an Infection System. *Methods Mol Biol*. 2016. doi:10.1007/7651_2016_7.
4. Hannan NRF, Fordham RP, Syed YA, et al. Generation of multipotent foregut stem cells from human pluripotent stem cells. *Stem cell reports*. 2013;1(4):293-306. doi:10.1016/j.stemcr.2013.09.003.
5. Forbester JL, Goulding D, Vallier L, et al. Interaction of Salmonella enterica Serovar Typhimurium with Intestinal Organoids Derived from Human Induced Pluripotent Stem Cells. *Infect Immun*. 2015;83(7):2926-34. doi:10.1128/IAI.00161-15.
6. Yusa K, Rashid ST, Strick-Marchand H, et al. Targeted gene correction of α1-antitrypsin deficiency in induced pluripotent stem cells. *Nature*. 2011;478(7369):391-394. doi:10.1038/nature10424.
7. Kraiczy J, Nayak K, Ross A, et al. Assessing DNA methylation in the developing human intestinal epithelium: potential link to inflammatory bowel disease. *Mucosal Immunol*. 2016;9(3):647-658. doi:10.1038/mi.2015.88.
8. Jenke AC, Postberg J, Raine T, et al. DNA Methylation Analysis in the Intestinal Epithelium-Effect of Cell Separation on Gene Expression and Methylation Profile. Eckle T, ed. *PLoS One*. 2013;8(2):e55636. doi:10.1371/journal.pone.0055636.
9. Aryee MJ, Jaffe AE, Corrada-Bravo H, et al. Minfi: a flexible and comprehensive Bioconductor package for the analysis of Infinium DNA methylation microarrays. *Bioinformatics*. 2014;30(10):1363-9. doi:10.1093/bioinformatics/btu049.
10. Du P, Zhang X, Huang C-C, et al. Comparison of Beta-value and M-value methods for quantifying methylation levels by microarray analysis. *BMC Bioinformatics*. 2010;11(1):587. doi:10.1186/1471-2105-11-587.
11. Leek JT, Johnson WE, Parker HS, Fertig EJ JA and SJ. sva: Surrogate Variable Analysis. *R Packag version 3180*.
12. GK S. limma. *Bioinforma Comput Biol Solut Using R Bioconductor*. 2005:397–420. Available at: http://bioconductor.org/packages/release/bioc/html/limma.html. Accessed November 7, 2014.
13. Martin M. Cutadapt removes adapter sequences from high-throughput sequencing reads. *EMBnet.journal*. 2011;17(1):10. doi:10.14806/ej.17.1.200.
14. Kim D, Pertea G, Trapnell C, Pimentel H, Kelley R, Salzberg SL. TopHat2: accurate alignment of transcriptomes in the presence of insertions, deletions and gene fusions. *Genome Biol*. 2013;14(4):R36. doi:10.1186/gb-2013-14-4-r36.
15. Langmead B. Aligning short sequencing reads with Bowtie. *Curr Protoc Bioinformatics*. 2010;Chapter 11:Unit 11.7. doi:10.1002/0471250953.bi1107s32.
16. Li H, Handsaker B, Wysoker A, et al. The Sequence Alignment/Map format and SAMtools. *Bioinformatics*. 2009;25(16):2078-9. doi:10.1093/bioinformatics/btp352.
17. Anders S, Pyl PT, Huber W. HTSeq - A Python framework to work with high-throughput sequencing data. *Bioinformatics*. 2014;31(2):166-169. doi:10.1093/bioinformatics/btu638.
18. Risso D, Ngai J, Speed TP, Dudoit S. Normalization of RNA-seq data using factor analysis of control genes or samples. *Nat Biotechnol*. 2014;32(9):896-902. doi:10.1038/nbt.2931.
19. Love MI, Huber W, Anders S. Moderated estimation of fold change and dispersion for RNA-seq data with DESeq2. *Genome Biol*. 2014;15(12):550. doi:10.1186/s13059-014-0550-8.
20. Zhao S, Guo Y, Sheng Q, Shyr Y. heatmap3: An Improved Heatmap Package. 2015. Available at: https://cran.r-project.org/web/packages/heatmap3/index.html.
21. Kim SK, Lund J, Kiraly M, et al. A gene expression map for Caenorhabditis elegans. *Science (80-)*. 2001;293(5537):2087-2092. doi:10.1126/science.1061603.

Supplementary Figures

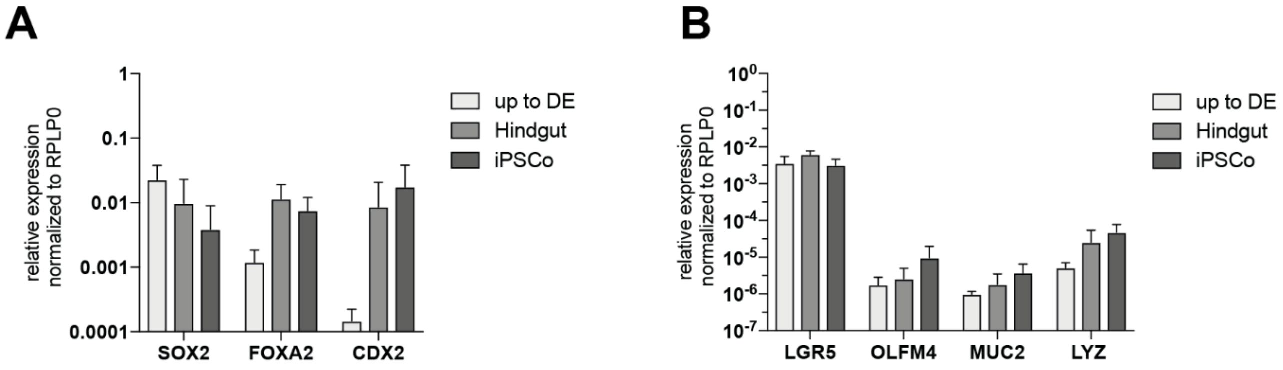

Supplementary Figure 1. Example real-time PCR data of (A) lineage markers and (B) intestinal epithelial markers over different stages of differentiation of human iPSCs, expressed as $\log_2$ fold change normalized to Ribosomal Protein Lateral Stalk Subunit P0 (RPLP0). Differentiation stages are summarized as the following: Up to definitive endoderm (DE)= Day 0 and 2; Hindgut= Day 6 and 8 , iPSCO= from day 10 on (3D culture). Data expressed as mean+SD, n=2-3 per group derived from one hiPSC line. *SOX2*= SRY-Box 2; *FOXOA2*= Forkhead Box A2; *CDX2*= Caudal Type Homeobox 2; *LGR5*= Leucine Rich Repeat Containing G Protein-Coupled Receptor 5; *OLFM4*= Olfactomedin 4; *MUC2*= Mucin 2; *LYZ*= Lysozyme.

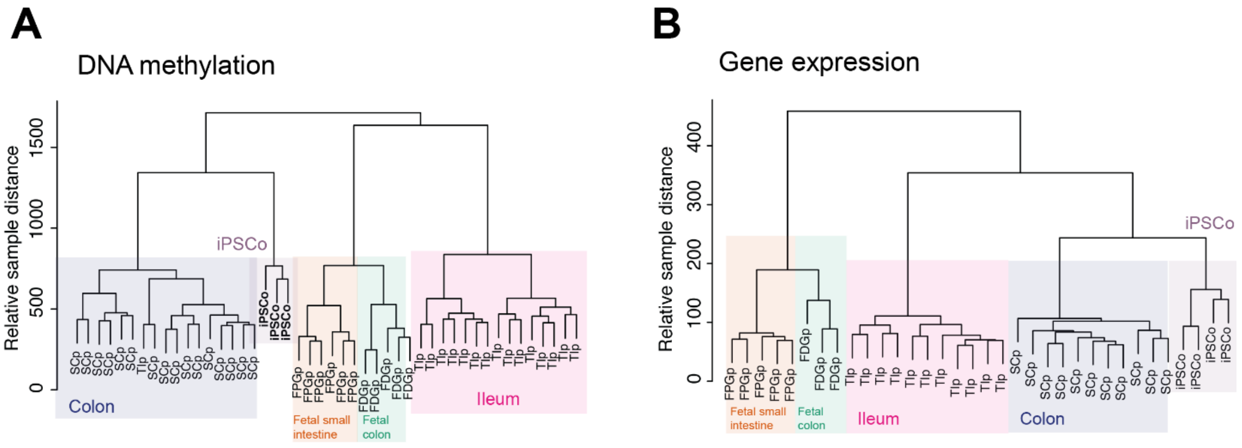

Supplementary Figure 2. Hierarchical clustering of sample relation based on (A) genome-wide DNA methylation levels and (B) RNA-sequencing profiles of intestinal epithelial organoids derived from iPSCs (iPSCo) as well as purified epithelial cells (EPCAM+) derived from pediatric terminal ileum (TIp), sigmoid colon (SCp) and fetal proximal gut (FPGp) or fetal distal gut (FDGp). Input data used were (A) M-values and (B) *rlog*-transformed counts of all genes.

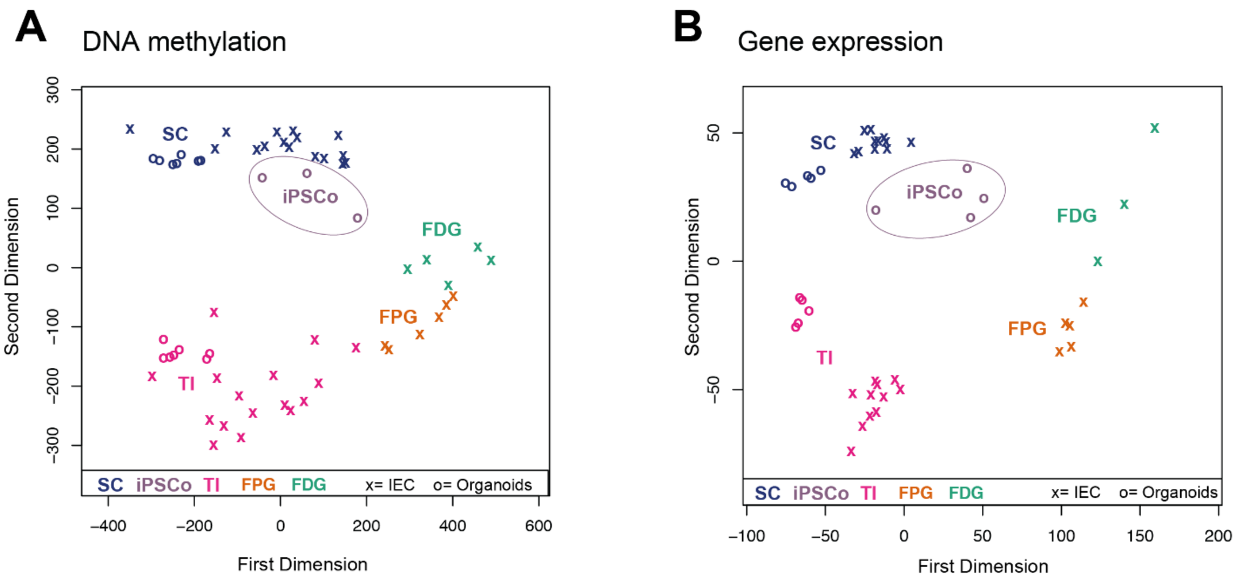

Supplementary Figure 3. Multidimensional scaling (MDS) of sample similarity based on (A) genome-wide DNA methylation levels (M-values) and (B) RNA-sequencing counts (*rlog*-transformed counts) for intestinal epithelial organoids derived from iPSCs (iPSCo) and mucosa-derived epithelial organoids from TI and SC (marked by “o”) in the context of purified epithelial cells (EPCAM+, marked by “x”) from TI, SC, FPG, FDG; see also Figure 2A and 2B.

Supplementary Table 1
Samples used for RNA-sequencing analysis

Type	Age group	Location	Patient	Age	Gender	Group
Organoids	Adult	iPSC	Kolf2	>18 yrs	M	iPSCo
Organoids	Adult	iPSC	Yemz1	>18 yrs	M	iPSCo
Organoids	Adult	iPSC	Lise1	>18 yrs	M	iPSCo
Organoids	Adult	iPSC	Bob/CA1ATD	>18 yrs	M	iPSCo
Purified	Paediatric	SC	1	13 yrs	M	SCp [1]
Purified	Paediatric	SC	7	14 yrs	F	SCp [1]
Purified	Paediatric	SC	15	15 yrs	F	SCp [1]
Purified	Paediatric	SC	22	14 yrs	F	SCp[1]
Purified	Paediatric	SC	23	14 yrs	M	SCp [1]
Purified	Paediatric	SC	31	9 yrs	M	SCp [1]
Purified	Paediatric	SC	39	14 yrs	M	SCp [1]
Purified	Paediatric	SC	42	15 yrs	M	SCp [1]
Purified	Paediatric	SC	56	13 yrs	F	SCp [1]
Purified	Paediatric	SC	63	6 yrs	M	SCp [1]
Purified	Paediatric	SC	71	9 yrs	M	SCp [1]
Purified	Pediatric	TI	1	13 yrs	M	TIp [1]
Purified	Paediatric	TI	7	14 yrs	F	TIp [1]
Purified	Paediatric	TI	15	15 yrs	F	TIp [1]
Purified	Paediatric	TI	22	14 yrs	F	TIp [1]
Purified	Paediatric	TI	23	14 yrs	M	TIp [1]
Purified	Paediatric	TI	31	9 yrs	M	TIp [1]
Purified	Paediatric	TI	39	14 yrs	M	TIp [1]
Purified	Paediatric	TI	42	15 yrs	M	TIp [1]
Purified	Paediatric	TI	56	13 yrs	F	TIp [1]
Purified	Paediatric	TI	63	6 yrs	M	TIp [1]
Purified	Paediatric	TI	71	9 yrs	M	TIp [1]
Purified	Fetal	FDG	FDGp1	8-12 wk GA	nk	FDGp [1]
Purified	Fetal	FDG	FDGp2	8-12 wk GA	nk	FDGp [1]
Purified	Fetal	FDG	FDGp3	8-12 wk GA	nk	FDGp [1]
Purified	Fetal	FPG	1792	8-12 wk GA	M	FPGp [1]
Purified	Fetal	FPG	FPG5	8-12 wk GA	nk	FPGp [1]
Purified	Fetal	FPG	FPG8	8-12 wk GA	nk	FPGp [1]
Purified	Fetal	FPG	Mr	8-12 wk GA	M	FPGp [1]
Purified	Fetal	FPG	Mg	8-12 wk GA	F	FPGp [1]
Organoids	Paediatric	SC	242	4 yrs	F	SCo [1]
Organoids	Paediatric	SC	212	11 yrs	M	SCo [1]
Organoids	Paediatric	SC	223	15 yrs	F	SCo [1]
Organoids	Paediatric	SC	224	15 yrs	F	SCo [1]
Organoids	Paediatric	SC	229	5 yrs	M	SCo [1]

nk= not known; yrs= years; wk GA= week in gestational age. [1] Data obtained from reference 1.

Supplementary Table 2

Samples used for DNA methylation analysis

Type	Age group	Location	Patient	Age	Gender	Group
Organoids	Adult	iPSC	Bob/CA1ATD	>18 yrs	M	iPSCo
Organoids	Adult	iPSC	Yemz1	>18 yrs	M	iPSCo
Organoids	Adult	iPSC	Kolf2	>18 yrs	M	iPSCo
Purified	Pediatric	SC	100	14 yrs	M	SCp [1]
Purified	Pediatric	SC	42	15 yrs	M	SCp [1]
Purified	Pediatric	SC	63	6 yrs	M	SCp [1]
Purified	Pediatric	SC	12	15 yrs	F	SCp [1]
Purified	Pediatric	SC	15	15 yrs	F	SCp [1]
Purified	Pediatric	SC	22	14 yrs	F	SCp [1]
Purified	Pediatric	SC	31	9 yrs	M	SCp [1]
Purified	Pediatric	SC	64	13 yrs	M	SCp [1]
Purified	Pediatric	SC	71	9 yrs	M	SCp [1]
Purified	Pediatric	SC	P_2	8 yrs	F	SCp [1]
Purified	Pediatric	SC	P_1	13 yrs	M	SCp [1]
Purified	Pediatric	SC	1	13 yrs	M	SCp [1]
Purified	Pediatric	SC	7	14 yrs	F	SCp [1]
Purified	Pediatric	SC	56	13 yrs	F	SCp [1]
Purified	Pediatric	SC	23	14 yrs	M	SCp [1]
Purified	Pediatric	SC	39	14 yrs	M	SCp [1]
Purified	Pediatric	TI	100	14 yrs	M	TIp [1]
Purified	Pediatric	TI	42	15 yrs	M	TIp [1]
Purified	Pediatric	TI	63	6 yrs	M	TIp [1]
Purified	Pediatric	TI	12	15 yrs	F	TIp [1]
Purified	Pediatric	TI	15	15 yrs	F	TIp [1]
Purified	Pediatric	TI	22	14 yrs	F	TIp [1]
Purified	Pediatric	TI	31	9 yrs	M	TIp [1]
Purified	Pediatric	TI	64	13 yrs	M	TIp [1]
Purified	Pediatric	TI	71	9 yrs	M	TIp [1]
Purified	Pediatric	TI	P_2	8 yrs	F	TIp [1]
Purified	Pediatric	TI	P_1	13 yrs	M	TIp [1]
Purified	Pediatric	TI	1	13 yrs	M	TIp [1]
Purified	Pediatric	TI	7	14 yrs	F	TIp [1]
Purified	Pediatric	TI	56	13 yrs	F	TIp [1]
Purified	Pediatric	TI	23	14 yrs	M	TIp [1]
Purified	Pediatric	TI	39	14 yrs	M	TIp [1]
Purified	Fetal	FDG	Mg	8-12 wk GA	F	FDGp [1]
Purified	Fetal	FDG	1627	8-12 wk GA	F	FDGp [1]
Purified	Fetal	FDG	F_2	8-12 wk GA	M	FDGp [1]
Purified	Fetal	FDG	F_3	8-12 wk GA	M	FDGp [1]
Purified	Fetal	FDG	F_1	8-12 wk GA	M	FDGp [1]
Purified	Fetal	FPG	1792	8-12 wk GA	M	FPGp [1]
Purified	Fetal	FPG	Mg	8-12 wk GA	F	FPGp [1]
Purified	Fetal	FPG	1690	8-12 wk GA	F	FPGp [1]
Purified	Fetal	FPG	F_1	8-12 wk GA	M	FPGp [1]
Purified	Fetal	FPG	F_2	8-12 wk GA	M	FPGp [1]
Purified	Fetal	FPG	F_3	8-12 wk GA	M	FPGp [1]

nk= not known; yrs= years; wk GA= week in gestational age. [1] Data obtained from reference 1.

Supplementary Table 3

Overlap statistics of differentially methylated positions and differentially expressed genes (Figure 2C)

	Total in dataset	Positions TI vs SC	Contrast	Positions	Overlap	Representation Factor	P-value
Differentially methylated positions (DMPs)	415,550	75,008	iPSCo vs TI	81,859	20,597	1.39	<1E-1000
			iPSCo vs SC	71,912	13,933	1.07	3.42E-24
Differentially expressed genes (DEGs)	24,467	8,808	iPSCo vs TI	6,208	3,842	1.72	<1E-1000
			iPSCo vs SC	4,347	2,335	1.49	2.93E-153
